# Evaluating the clinical impact of routine whole genome sequencing in tuberculosis treatment decisions and the issue of isoniazid mono-resistance

**DOI:** 10.1186/s12879-022-07329-y

**Published:** 2022-04-07

**Authors:** Mirae Park, Ajit Lalvani, Giovanni Satta, Onn Min Kon

**Affiliations:** 1 Department of Respiratory Medicine, Imperial College Healthcare NHS Trust, St Mary’s Hospital, Praed Street, London, UK; 2 National Heart and Lung Institute, Faculty of Medicine, Imperial College London, London, SW3 6LY UK; 3 Tuberculosis Research Centre, National Heart and Lung Institute, Imperial College London, Norfolk Place, London, W2 1PG UK; 4 Department of Infectious Diseases, Imperial College Healthcare NHS Trust, St Mary’s Hospital, Praed Street, London, W2 1NY UK

**Keywords:** Whole genome sequencing, Tuberculosis, Treatment alteration, Turnaround times, Isoniazid mono-resistance

## Abstract

**Background:**

The UK has implemented routine use of whole genome sequencing (WGS) in TB diagnostics. The WHO recommends addition of a fluoroquinolone for isoniazid mono-resistance, so early detection may be of use. The aim of this study was to describe the clinical utility and impact of WGS on treatment decisions for TB in a low incidence high resource clinical setting. The clinical turnaround time (TAT) for WGS was analysed in comparison to TB PCR using Xpert MTB/RIF (Cepheid, Sunnyvale, CA) results where available and subsequent phenotypic drug susceptibility testing (DST) when required.

**Methods:**

This was a retrospective analysis of TB cases from January 2018 to March 2019 in London. Susceptibility and TAT by WGS, phenotypic DST, TB PCR using Xpert MTB/RIF were correlated to drug changes in order to describe the utility of WGS on treatment decisions on isoniazid mono-resistance in a low incidence high resource setting.

**Results:**

189 TB cases were identified; median age 44 years (IQR 28–60), m:f ratio 112:77, 7 with HIV and 6 with previous TB. 80/189 cases had a positive culture and WGS result. 50/80 were fully sensitive to 1st line treatment on WGS, and the rest required additional DST. 20/80 cases required drug changes; 12 were defined by WGS: 8 cases had isoniazid mono-resistance, 2 had MDR-TB, 1 had isoniazid and pyrazinamide resistance and 1 had ethambutol resistance. The median TAT for positive culture was 16 days (IQR 12.5–20.5); for WGS was 35 days (IQR 29.5–38.75) and for subsequent DST was 86 days (IQR 69.5–96.75), resulting in non-WHO regimens for a median of 50.5 days (IQR 28.0–65.0). 9/12 has TB PCRs (Xpert MTB/RIF), with a median TAT of 1 day.

**Conclusion:**

WGS clearly has a substantial role in our routine UK clinical settings with faster turnaround times in comparison to phenotypic DST. However, the majority of treatment changes defined by WGS were related to isoniazid resistance and given the 1 month TAT for WGS, it would be preferable to identify isoniazid resistance more quickly. Therefore if resources allow, diagnostic pathways should be optimised by parallel use of WGS and new molecular tests to rapidly identify isoniazid resistance in addition to rifampicin resistance and to minimise delays in starting WHO isoniazid resistance treatment.

## Background

Tuberculosis (TB) is a communicable disease caused by the bacillus *Mycobacterium tuberculosis* (MTB)*.* It remains a major global health burden with an estimated 10 million cases in 2019 [1]. TB also remains in the top ten causes of death worldwide, responsible for over 1.4 million deaths per year [1]. There remains a gap of 2.9 million between the notified and estimate cases, partly due to a combination of underreporting and underdiagnosing. Despite a fall in the incidence over the last few years, there is a growing issue of drug resistant TB. Drug resistant TB not only requires confirmation and culture of the bacteria but also drug susceptibility testing (DST).

Diagnostics in TB have accelerated in the last few years with the accessibility to molecular techniques and the introduction of whole genome sequencing (WGS). WGS enables the complete DNA sequencing of MTB allowing for not only detection, but also for complete genotypic drug susceptibilities as well as transmission data [2–4]. The UK was the first country to implement routine use of WGS and adopt a national network to optimise TB control and for the primary diagnostic tool of MTB detection [5]. There have been several studies to show the scalability, rapid turnaround time (TAT) and financial feasibility for WGS [6–8]. This is in addition to the correlation of genotypic prediction of the susceptibility of MTB to first line TB treatment with phenotypic drug susceptibilities [6, 9]. A clinical study in a UK demonstrated the median TAT from sample receipt in the reference laboratory to identification of MTB species to be 6 week days and results of DST to be between 8 to 12 days [10]. The UK WGS model has been shown to be exportable to other low incidence countries. When implemented in Italy, WGS results were available within 72 h of delivery of the culture sample whilst phenotypic drug susceptibility testing (pDST) for first line treatment took 28 days after culture confirmation [11]. The use of WGS on diagnosis of drug resistant TB and choices of appropriate treatment in high burden settings have been demonstrated [12], and in the UK, the clinical application of WGS has been shown by using WGS to analyse sixteen isolates to identify genotypic mutations which may predict potential antibiotic resistance [8]. There are also retrospective studies to compare the impact of phenotypic and molecular drug resistance testing on therapy for drug resistant TB with Xpert MTB/RIF (Cepheid, Sunnyvale, CA) having a 49% agreement in the number of drugs prescribed compared to pDST. This was higher for line probe assays (LPA) (Hain GenoType MTBDR*plus* 2.0 and MTBDR*sl*2.0) at 79% and the agreement was 93% for WGS [13]. Next-generation sequencing (NGS) has also been shown to reliably guide the design of effective multidrug resistant TB (MDR-TB) drug regiments [14]. However there has not yet been a real time clinical study to describe the impact of WGS on front line decision making for alterations in TB drug treatment in a low burden setting.

With growing concerns for drug resistant TB, including the rise in isoniazid mono-resistance and MDR-TB, rapid identification of TB as well as the drug susceptibilities are essential [15]. The World Health Organisation (WHO) recommendation of isoniazid mono-resistance treatment now involves the addition of a fluoroquinolone [16], so early detection of isoniazid resistance may be of use.

## Objectives

The objective of this study was to describe the clinical utility and impact of WGS on treatment decisions for TB in a low incidence high resource clinical setting. Any changes to TB treatment related to WGS results were identified and analysed. The TAT for WGS was analysed in comparison to TB PCR (Xpert MTB/RIF) results where available and subsequent pDST when required.

## Methods

This was a retrospective data analysis of 189 consecutively registered pulmonary and extra-pulmonary TB cases from January 2018 to March 2019 in a tertiary TB Centre in London, UK. Processed specimens were inoculated into a MGIT tube, placed in the Bactec MGIT 960 system for continuous monitoring until positive or until the end of the testing protocol. Since 2018, WGS has been performed on all MTB culture positive samples as part of the routine clinical diagnostic service in a centralised national laboratory. All methods were carried out in accordance with relevant guidelines and regulations.

Patient demographics, clinical phenotypes including TB risk factors, and follow up data were collected from patient medical records and the London TB Registry. TB cases were confirmed clinically. The TAT was calculated from sample collection until availability of results which were reviewed from the laboratory pathology systems (where available) and correlated to the alterations made in TB drug regimens. For WGS and pDST, the TAT from sample collection included the initial days to culture positivity. Phenotypic DST was performed after WGS testing if there were any invalid (failed or unknown) results or any drug resistance identified on WGS. Xpert MTB/RIF was the only TB PCR method used.

Statistical analysis was performed on PRISM using Wilcoxon test for categorical variables with the level of significance set to α = 0.05.

Imperial College Research Ethics Committee, the local research governance ethics committee approved the criteria that ethical clearance was not required as there was no experimental protocols involved, there was no modification to the routine laboratory workflow or the clinical patient management and hence no informed consent was required.

## Results

**Fig. 1:**
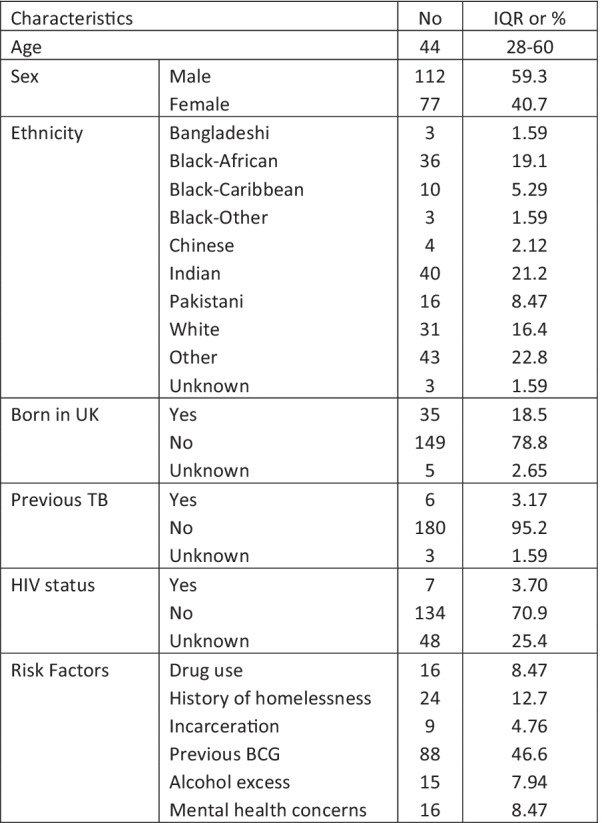
Summary of demographics for the study population with details of sex, ethnicity, HIV status and risk factors for TB

One hundred and eighty-nine TB cases were identified from the London TB registry, all clinically diagnosed with TB during January 2018 to March 2019. The median age was 44 years (IQR 28–60). The male to female ratio was 112:77, 7 patients had known HIV, and 6 with previous TB. Demographic data are shown in Fig. 1.

From the 189 cases initially started on TB treatment, 25 had their treatment stopped due to a change in clinical diagnosis. Of the remaining 164 cases, 57 were culture negative, 86 cases were culture positive and the rest had missing data points. There were 6 culture positive cases with missing WGS data or issues with WGS as such contamination of samples.

**Fig. 2:**
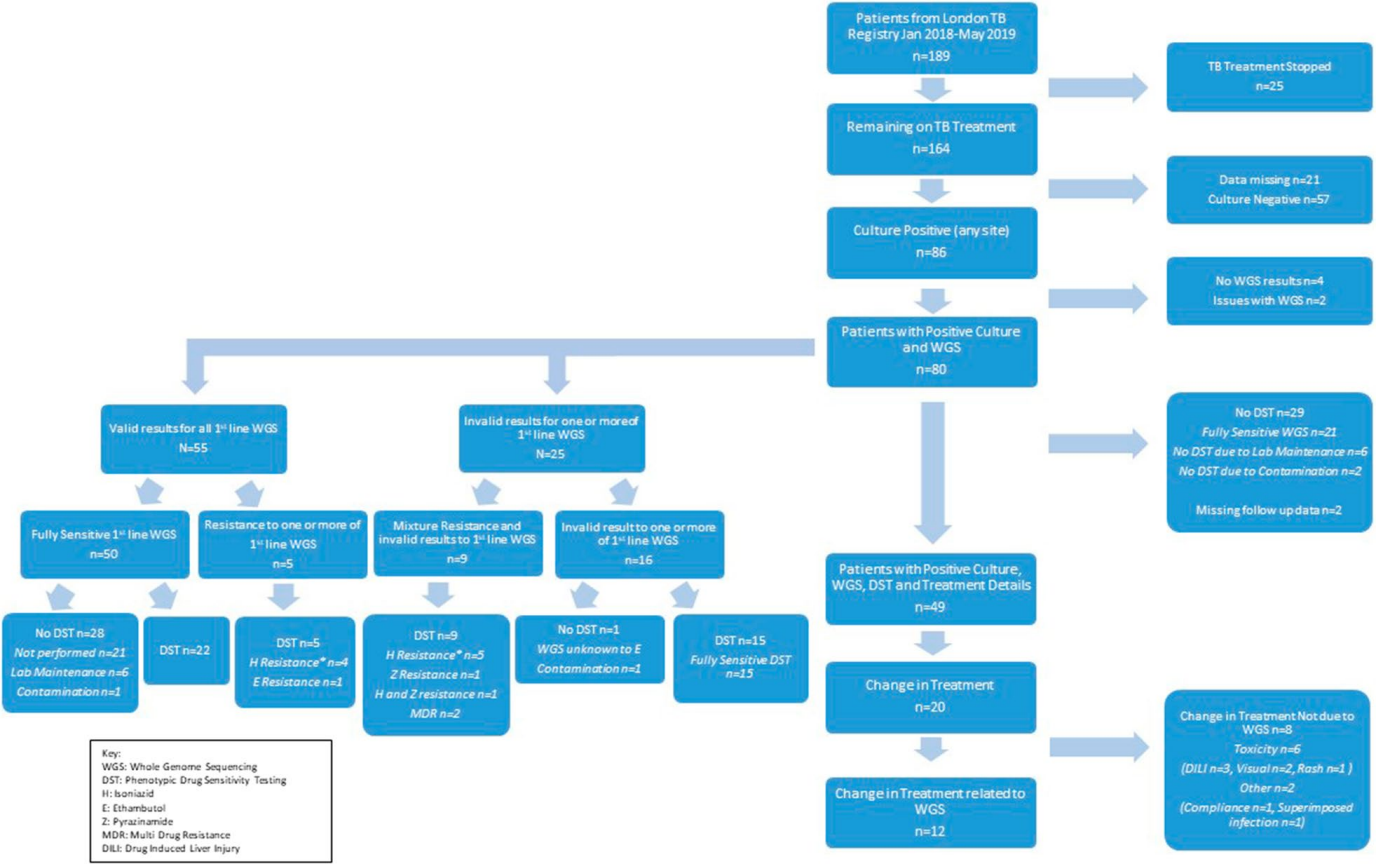
Study flow chart with 189 cases initially being identified from the London TB registry of which 80 cases had positive culture and WGS results. Twenty cases required treatment change of which 12 were related to the WGS results and 8 were unrelated to WGS results. The left side of the figure shows the breakdown of cases with valid or invalid (failed or unknown) 1st line WGS results

Forty-nine out of the 80 cases (with positive culture and WGS results), also had pDST and full treatment details. During the study period, Public Health England (PHE) approved a new laboratory workflow where no pDST was performed on samples fully susceptible to first line drugs on WGS. Twenty-nine cases did not have pDST testing of which 21 were fully susceptible on WGS, 8 had processing issues of which 1 case had an invalid result to Ethambutol on WGS. Please see Fig. 2 for a summary of the study flow chart.

### WGS results

From the 80 cases with positive culture and WGS results, 55 cases had valid susceptibility results for all first line drug treatment (rifampicin (R), isoniazid (H), pyrazinamide (Z) and ethambutol (E)) on WGS. Fifty cases were fully sensitive to first line drug treatment on WGS and the remaining 5 cases were resistant to one or more of the first line drug treatment. Twenty-five cases had an invalid (failed or unknown) result to one or more of the first line drugs on WGS. Of these, 9 cases also had concurrent resistance patterns to one or more of the first line drugs; 5 cases with invalid results to rifampicin resistance, of which 4 were in isoniazid resistant cases. One case was resistant to isoniazid but had an invalid result for ethambutol and pyrazinamide. All 9 cases had pDST completing the sensitivity profile.

Only 1 case had invalid results for first line drug susceptibility on WGS (unknown ethambutol resistance pattern) and a failed pDST result due to fungal contamination of the culture. Fortunately, in this case the patient was initially started on RHZ with moxifloxacin (M) due to ocular TB disease, hence the drug treatment was not altered.

The TAT for all WGS results (n = 80) from sample collection was 34 days (IQR 28–38). The TATs for the 25 cases with invalid results to one or more of the first line drugs on WGS was 35 days (IQR 24–40) and 75 days (IQR 63–91.5) for the subsequent pDST (from sample collection and including the initial TAT for WGS). The TAT was similar for the cases with valid results for 1^st^ line drug susceptibilities on WGS taking 32 days (IQR 28–43.5) and subsequent pDST taking 74 days (IQR 67.5–85.5) and hence not significantly different (TAT for invalid vs valid WGS p = 0.723; TAT for invalid vs valid subsequent DST p = 0.354).

### Treatment alterations related to WGS results

**Fig. 3:**
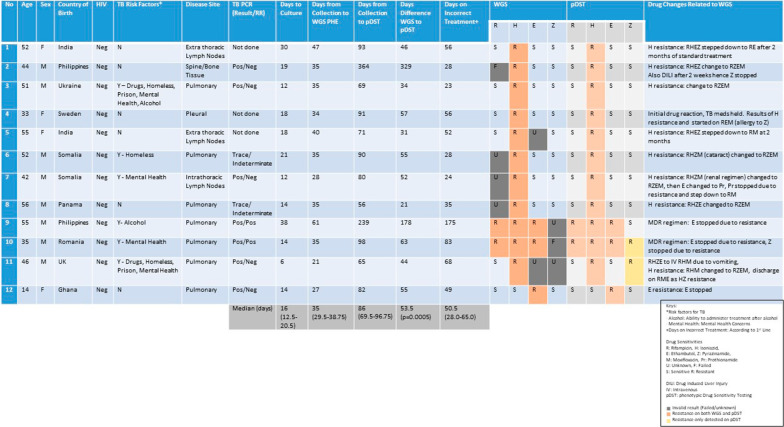
Summary of the 12 cases that required drug treatment changes defined by WGS results. Eight cases were as a result of isoniazid mono-resistance, 2 cases of MDR-TB, 1 case of isoniazid and pyrazinamide resistance and 1 case of ethambutol resistance. The median TATs are shown on the bottom row

Twenty out of the 80 cases (which had both positive culture and WGS results) had alterations to TB treatment regimens. However, 12 out of 20 cases (15% of the original 80 cases) had treatment changes defined by WGS, summarized in Fig. 3. The commonest resistance pattern identified on WGS was isoniazid mono-resistance in 8 cases, leading to isoniazid being stopped and moxifloxacin being added in to the treatment regimen. Two cases had complete drug alterations due to MDR-TB (details under MDR-TB). One case was resistant to isoniazid and pyrazinamide and one case had ethambutol mono-resistance leading to appropriate treatment adjustments.

**Fig. 4:**
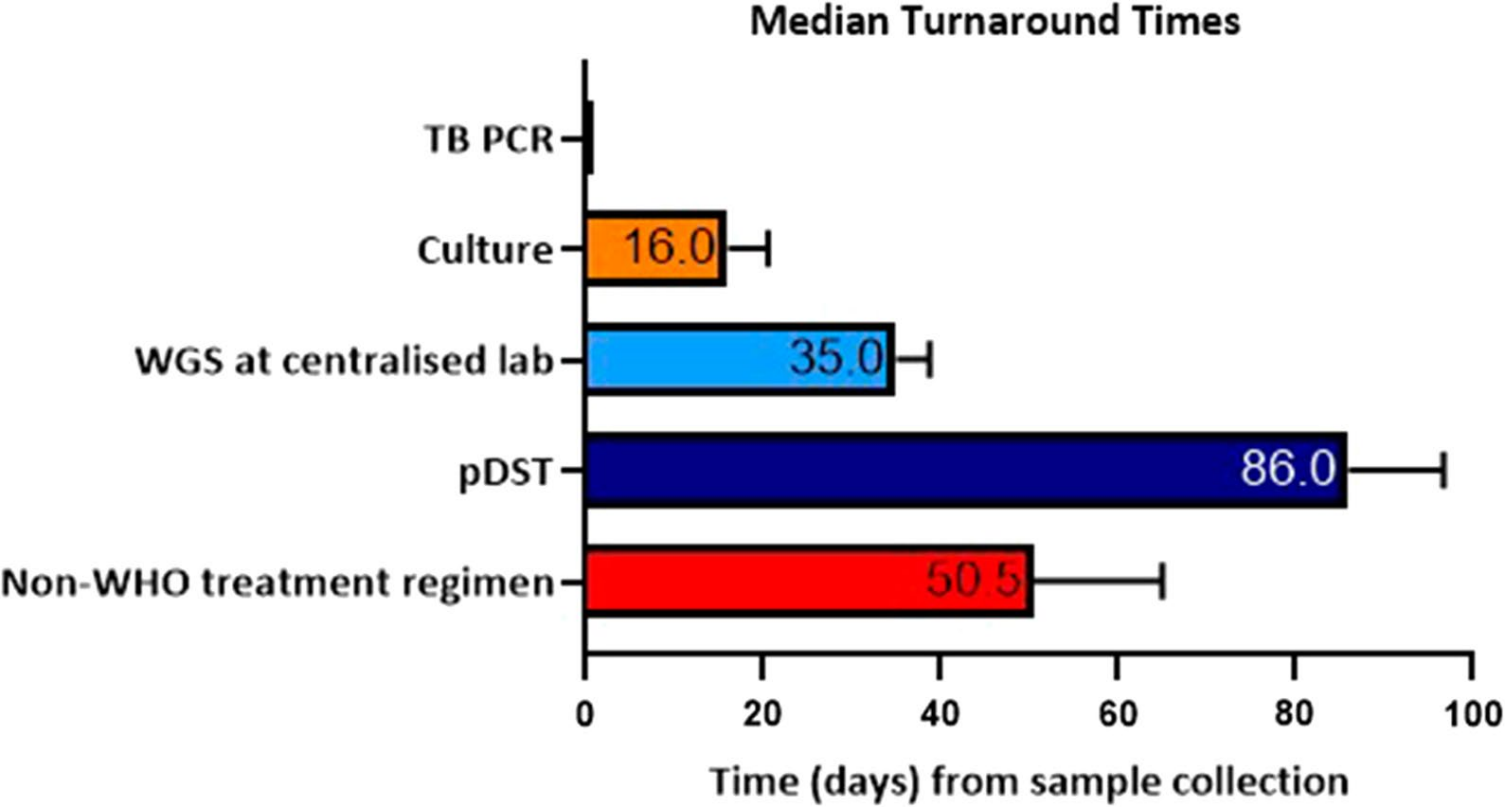
Median TATs from sample collection for different TB diagnostic tests for the 12 cases that required treatment change as a result of WGS. WGS results at a central lab took a median of 35 days and additional pDST took 86 days from sample collection

For these 12 cases, the median time for culture positivity was 16 days (IQR 12.5–20.5). Median TAT from samples collection to complete WGS results in the centralised laboratory at PHE was 35 days (IQR 29.5–38.75). The median TAT from sample collection to WGS results to be uploaded on electronic medical records was 97.5 days (72.0–114.8). The median TAT from sample collection to additional pDST results (after initial WGS result) was 86 days (IQR 69.5–96.75), resulting in a median delay of 53.5 days (p = 0.0005) between the WGS and pDST results. When the initial TAT for WGS is accounted for, DST still had a significantly longer TAT compared to WGS with a median TAT delay of 23 days (IQR 12.75–28) (p < 0.019). The median time from initial treatment start to establishing the correct treatment regimen was 50.5 days (IQR 28.0–65.0); meaning patients were on a non-WHO treatment regimen for over a month. These data are summarised in Fig. 4.

### Isoniazid resistance

Twelve cases out of the 80 culture positive cases with WGS results had resistant mutations to isoniazid, which included the 2 cases of MDR-TB, 9 cases of isoniazid mono-resistance (with one case lost to follow-up) and 1 further case of isoniazid resistance and pyrazinamide resistance later identified on pDST. Three cases had invalid results for isoniazid resistance on WGS for but subsequently found to be sensitive pDST. Eight of 12 cases that had treatment changes defined by WGS were related to isoniazid mono-resistance.

### Treatment alterations not related to WGS results

**Fig. 5:**
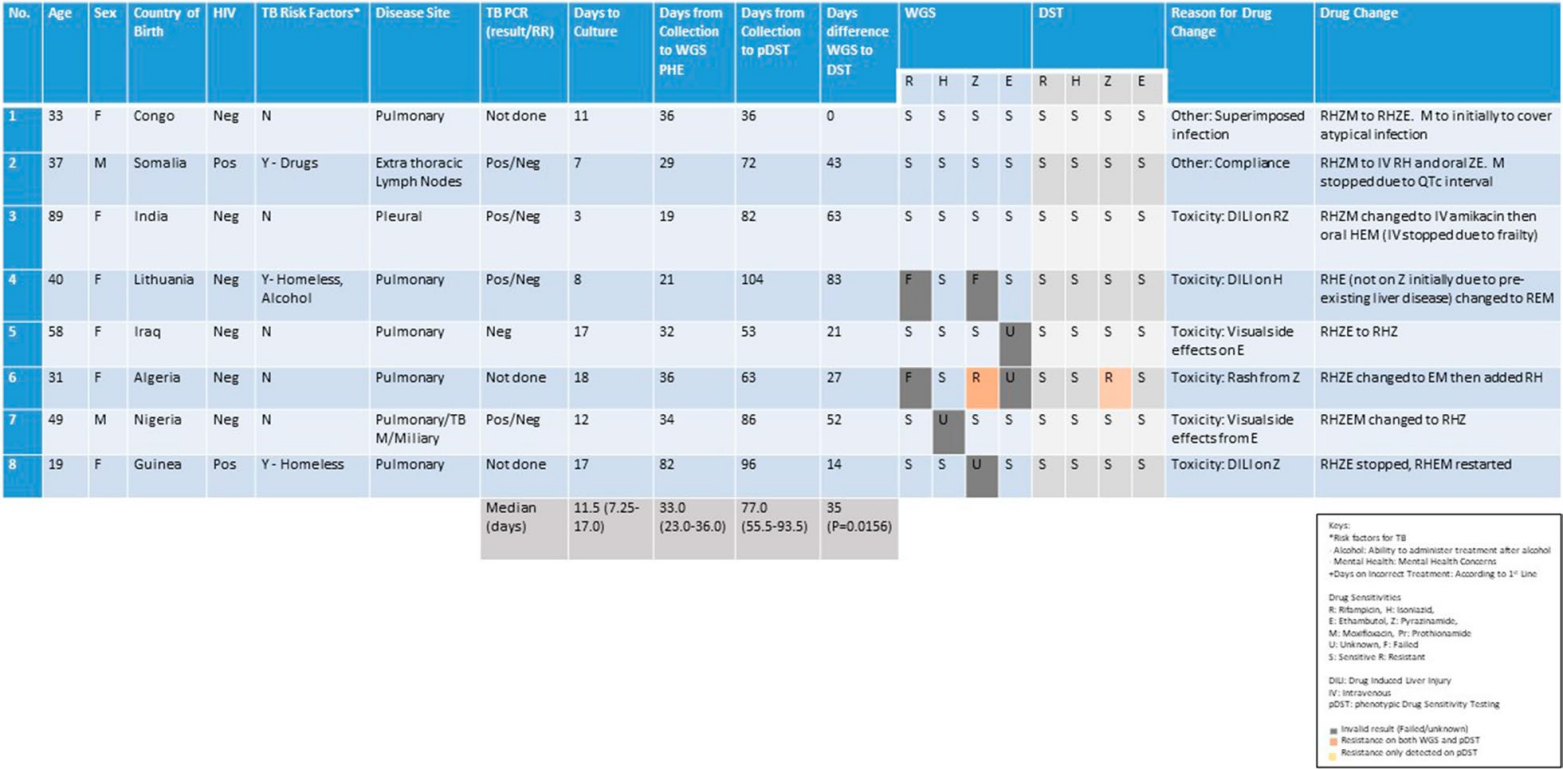
Summary of 8 cases that required drug treatment changes that were not related to WGS results. These included 3 cases of drug induced liver injury, 2 cases of ethambutol toxicity, 1 case of pyrazinamide rash, 1 case of non-compliance and a case where drug alterations were made for clinical reasons

There were 8 cases (10% of the original 80 cases with positive culture and WGS) where treatment alterations were not directly related to WGS results. The commonest pattern was due to toxicities and side effects from TB treatment with 3 cases of drug induced liver injury (2 cases from pyrazinamide, 1 case from isoniazid). There were 2 cases of ethambutol toxicity with reported visual changes leading to the cessation of ethambutol use. The other 2 cases needing treatment alterations were from compliance issues hence changing from oral to intravenous preparations and one case where the initial regimen included moxifloxacin to cover superimposed infections. Details of these are summarised on Fig. 5.

### *TB PCR* (Xpert MTB/RIF)

Sixty-three of the 80 culture positive cases with WGS results had an initial TB PCR test performed prior to the culture result becoming available, of which 50 cases were PCR positive and detected MTB. These included the 2 MDR-TB with rifampicin resistance being identified. There were 12 out of 80 cases with an invalid result for rifampicin resistance on WGS but in 9 of these cases, TB PCR determined sensitivity to rifampicin. In the 12 cases with invalid rifampicin resistance on WGS, there were 2 ‘Trace’ readings (hence no resistance pattern available for rifampicin) and 1 case where TB PCR was not performed but was sensitive to rifampicin on pDST, 63 days after collection of samples. All together there were 5 cases showing ‘Trace’ results in the culture positive cases, all of which were in non-sputum samples (3 bronchial alveolar lavage, 1 mediastinal and 1 vertebral aspirate).

Of the 12 cases that had treatment alterations defined by WGS, 9 TB PCR results were available rapidly determining TB diagnosis and rifampicin resistance with a median TAT within 24 h of sample collection. Treatment was either started or adjusted on the basis of the TB PCR results, with further modification to the treatment regimen with the results of WGS, stopping of isoniazid in 6 of these cases, and stopping of ethambutol in 1 case. TB PCR identified both the MDR-TB cases which are discussed below.

### MDR-TB

Both the MDR-TB cases were rapidly detected using TB PCR with identification of rifampicin resistance acting as a surrogate marker for MDR-TB, hence changing the standard treatment regimen completely. There were further changes to the treatment regimen as a result of WGS results. Both cases were resistant to ethambutol and one case was resistant to pyrazinamide, resulting in stopping of these drugs and adjusting the regimen. The TAT from sample collection to WGS results in a centralised lab was 61 days for case 1 and 35 days for case 2. There was a further delay of 178 and 63 days respectively for case 1 and 2 for the availability of pDST results from the WGS results.

## Discussion

This study reports the useful implementation of WGS in routine clinical practice in a low incidence high resourced setting. WGS results were available in a centralised laboratory 35 days from sample collection (including the time to culture MTB first) which was 53.5 days before availability of the subsequent pDST results. This meant that genotypic sensitivities were available at the time of treatment stepdown from the intensive to continuation treatment phase at 2 months.

The median TAT for pDST (for the 12 cases that had treatment changes as a result of WGS) was 86 days (IQR 69.5–96.75) from collection, and 72 days (IQR 53.75–95.25) from treatment initiation, resulting in the pDST only being available after the 2 months mark, affecting the potential stepdown of medications. There were 2 cases where the pDST results were significantly delayed due to lab processing and recording errors, but even with these cases excluded the median TAT from sample collection to pDST results were similar at 81 days (IQR 68.0–91.5).

Further evaluation the TATs in a clinical setting, there was in fact a significant delay for the WGS results to be relayed into the medical notes with a median TAT of 97.5 days, when in fact the results were available in the central laboratory much earlier. This is a potential risk of the consolidation of pathology laboratories [17] but in our study, this appears to be more related to administrative and logistical issues rather than the implementation of this new technology. Such delay was not reflected in clinical practice as this did not correlate with delayed treatment alterations and alternative communication methods between the centralised lab and the treating physician (bypassing the local laboratory) were present. Compared to other historic studies where the TAT for WGS drug susceptibility results took within 72 h of sample receipt in Italy [11] and between 8 to 12 days in the UK [10], the TAT for WGS was longer but the results of this study importantly reflected a routine clinical setting by studying the real impact to clinicians by taking into account sample collection, sample transportation locally, time taken to culture MTB, transportation to a centralised lab and processing of the samples at a time when the WGS system was relatively newly established. There was also a significant delay to the pDST results being available by using the same time point criteria.

WGS is clearly significantly faster than pDST but the availability of the results still took over a month from sample collection. This stresses the importance of trying to improve not only the speed of WGS analytical processing but also the pathway of communication of the results to frontline clinicians. It is also important to note that WGS still requires a positive culture result and this will be a potential area to develop in the future with WGS being performed directly from samples to enhance the TAT. Some studies have already analysed WGS from sputum samples directly [18–20]. Another issue to note is the lack of positive culture results for around 35% of patients treated as suspected TB (57 out of 164). Previous data on culture negative TB varies from 15 to 37%, highlighting the limitations of current culture methods that can inevitably affect the availability of WGS results [21]. Another issue was the high rates (31%) of invalid results to one or more of the first line drugs on WGS. This was significantly higher compared to other studies reporting a 9% rate of WGS failure [11]. This could be explained by numerous factors such as insufficient DNA quantity for WGS analysis as well as initial glitches in a newly established pathway. Laboratories should carefully consider the percentage of WGS failures and resistance rates before completely switching to a WGS based system to avoid additional delays in setting up DST.

Even with the optimisation of the WGS pathway, one key issue this study highlighted was the delayed identification of isoniazid resistance. Excluding the 2 MDR-TB cases, 9 out of the 12 cases that had treatment changes as a result of WGS were due to isoniazid resistance (8 cases of isoniazid mono-resistance). Despite the WGS results, the median TAT was 35 days at the earliest point in a centralised laboratory, hence isoniazid resistance was not identified until then; ultimately leading to patients on a non-WHO treatment regimen for a median of 50.5 days (IQR 28.0–65.0). TB PCR testing was performed for most of these cases, rapidly confirming TB or MDR-TB with identification of rifampicin resistance. Even if rifampicin resistance was not detected at the point of care, fully sensitive TB could not be assumed. A known limitation of the commonly used Xpert MTB/RIF platform is its inability to detect isoniazid single resistance, only relying on the *rpoB* gene as surrogate marker of multidrug resistance [22].

Given the growing issue of isoniazid resistance, there is a clear need for development of extended panel of sensitivities to also identify isoniazid resistance in addition to rifampicin resistance. Currently there are several commercial LPAs which are able to detect rifampicin and isoniazid resistance mutations such as Nipro NTM + MDRTB (Tokyo, Japan) and GenoType MDRTB*plus* (Hain, Lifescience, Nehren, Germany) which are now WHO approved and are suitable for being performed in a central laboratory. However LPAs require post PCR manipulation and are not routinely used in local laboratories, even in high resource settings. These are currently only recommended in smear positive specimens and culture isolates of MTB [23]. Other commercial platforms and *inhouse* multiplex PCR are available [24–26] but again not commonly used in the UK.

Next-generation sequencing also has a potential role in the rapid diagnosis of drug resistant TB and can overcome limitations of less comprehensive molecular tests by providing detailed sequence information for multiple gene regions of interest [27]. There have been numerous studies to support the use of NGS and the use of a portable sequencing platform directly from clinical samples [14, 28, 29]. Despite concerns with cost, technical skillsets required and integration into current laboratory workflow, NGS shows great potential in advancing TB diagnostics. The WHO has recently updated their guidelines on molecular diagnostics and have strongly advocated for early diagnosis and universal access to DST especially to rifampicin, isoniazid and fluoroquinolones testing, classifying rapid tests according to the complexity of the test for implementation [27, 30].

One of the new molecular tests discussed in this guideline is the recently released Xpert MTB/XDR (Cepheid, Sunnyvale, USA). This is currently the only test under the low complexity category and allows testing for isoniazid, fluoroquinolones and second line injectable drugs (amikacin, kanamycin, capreomycin) and ethionamide. Other automated nucleic acid amplification tests (NAAT) evaluated in the WHO guidelines under a moderate complexity category include Abbott RealTi*me* MTB and MTB RIF/INH (Abbott Laboratories, Abbott Park, USA), BD MAX MDR-TB (Becton, Dickinson and Company, Franklin Lakes, USA), Hain FluroType MTBDR (Bruker/Hain Lifescience, Nehren, Germany) and Roche cobas MTB and MTB-RIF/INH (Hoffmann-La Roche, Basel, Switzerland).

With the amplification of TB DNA, NAAT technologies may additionally pick up smear negative cases or paucibacillary disease and may help identify drug resistance in the culture negative cases [5], a limitation identified in this study as all WGS are currently only performed on culture positive samples.

Interestingly there was only 1 case resistant to fluoroquinolones on WGS but this case was sensitive to the first line treatment. Of the isoniazid mono-resistant cases, there were 4 cases with unknown resistance to fluoroquinolones. Given the current WHO treatment guidance for isoniazid mono-resistance with the addition on a fluoroquinolone, there are certain situations where both WGS and pDST results are beneficial, especially if this will allow for a shorter treatment duration and reduce unnecessary drug toxicity side effects. Given our data, in a high resource low burden setting, TB PCR, WGS and pDST seem to be clinically indicated to ensure an optimal treatment regimen and potentially patient outcome.

This study had several limitations beyond its retrospective nature. As the study was set in a low incidence setting, despite reviewing the data for over a year the sample size was small with even fewer culture positivity rates. TB patients were identified from the London TB registry; a database based on clinical diagnosis of TB rather than just culture positive cases and including cases of extra-pulmonary disease, which is more paucibacillary in nature hence having lower culture positivity rates.

During this study period, PHE set a new workflow pathway where pDST was only performed when resistance to first line drugs were detected genotypically. This led to a small sample size for comparing WGS and pDST in this study.

As this study was set in a high resource low burden setting, there needs to be a similar study in a low resource high burden setting, in low and middle income countries where drug resistance is a growing concern. The implication and clinical usefulness of WGS needs to be analysed to highlight the potential differences.

## Conclusion

In summary, WGS clearly has a substantial role in our routine UK clinical settings with faster turnaround times in comparison to pDST. This is in addition to its known role in data transmission and outbreak detection.

**Fig. 6:**
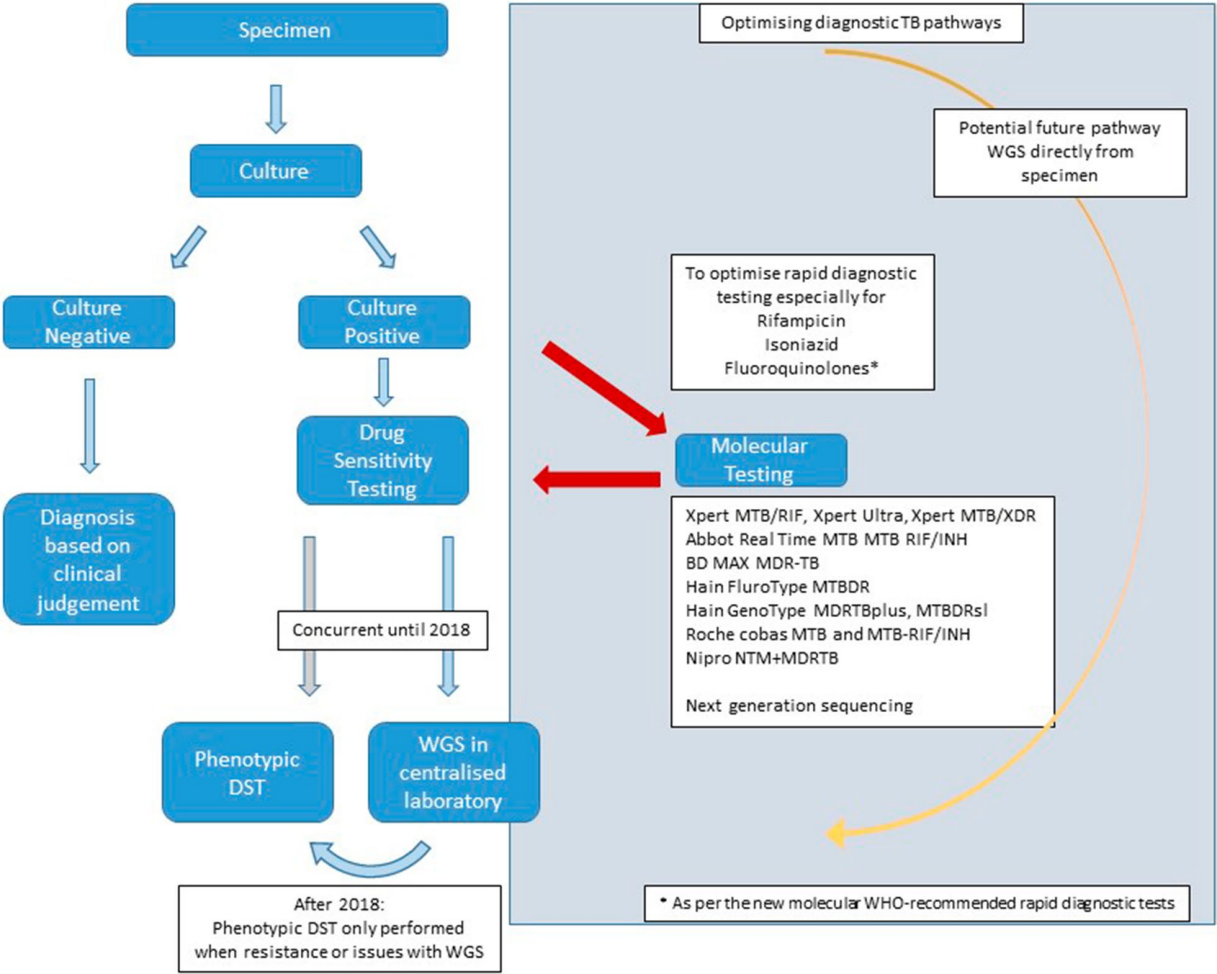
Current TB diagnostic pathway (left) and potential diagnostic pathways to optimise rapid diagnosis (right) including WGS and additional molecular testing if resources allow

However, the majority of treatment changes defined by WGS were related to isoniazid resistance and given the 1 month TAT for WGS at its earliest point, it would be preferable to identify isoniazid resistance more quickly. Where resources allow, a solution to this clinical problem identified in this study is to use commercially available LPAs or PCR techniques such as Xpert MTB/XDR in parallel with WGS in order to rapidly identify isoniazid resistance (in addition to rifampicin) within one to two days. The current and potential diagnostic pathways are shown in Fig. 6.

As a complementary diagnostic tool to WGS, point of care testing for the early identification of isoniazid resistance may have a significant impact on immediate treatment choices and minimise the number of days on non-WHO treatment regimens.

## Data Availability

The datasets used and/or analysed during the current study are available from the corresponding author on reasonable request.

## Abbreviations

TB: Tuberculosis
MTB: *Mycobacterium tuberculosis*
WGS: Whole genome sequencing
TAT: Turnaround time
MDR: Multidrug resistant
WHO: World Health Organisation
PCR: Polymerase Chain Reaction
pDST: Phenotypic drug susceptibility testing
PHE: Public Health England
R: Rifampicin
H: Isoniazid
Z: Pyrazinamide
E: Ethambutol
M: Moxifloxacin
NAAT: Nucleic acid amplification tests
LPA: Line probe assay
NGS: Next-generation sequencing

## References

[1] GLOBAL TUBERCULOSIS REPORT 2020. 2020. http://apps.who.int/bookorders. Accessed 11 Dec 2020

[2] Walker TM, Kohl TA, Omar SV, Hedge J, Elias CDO, Bradley P (2015). Whole-genome sequencing for prediction of *Mycobacterium tuberculosis* drug susceptibility and resistance: a retrospective cohort study. Lancet Infect Dis.

[3] Walker TM, Kohl TA, Omar SV, Hedge J, Elias CD, Bradley P, Iqbal Z, Feuerriegel S, Niehaus KE, Wilson DJ, Clifton DA (2015). Whole-genome sequencing for prediction of *Mycobacterium tuberculosis* drug susceptibility and resistance: a retrospective cohort study. Lancet Infect Dis.

[4] Gardy JL, Johnston JC, Sui SJH, Cook VJ, Shah L, Brodkin E (2011). Whole-genome sequencing and social-network analysis of a tuberculosis outbreak. Lancet Infect Dis.

[5] Satta G, Lipman M, Smith GP, Arnold C, Kon OM, McHugh TD (2017). *Mycobacterium tuberculosi*s and whole-genome sequencing: how close are we to unleash its full potential?. Clin Microbiol Infect.

[6] Pankhurst LJ, Del Ojo EC, Votintseva AA, Walker TM, Cole K, Davies J (2016). Rapid, comprehensive, and affordable mycobacterial diagnosis with whole-genome sequencing: a prospective study. Lancet Respir Med.

[7] Cirillo DM, Cabibbe AM, De Filippo MR, Trovato A, Simonetti T, Rossolini GM (2016). Use of WGS in *Mycobacterium tuberculosis* routine diagnosis. Int J Mycobacteriol.

[8] Witney AA, Gould KA, Arnold A, Coleman D, Delgado R, Dhillon J (2015). Clinical application of whole-genome sequencing to inform treatment for multidrug-resistant tuberculosis cases. J Clin Microbiol.

[9] The CRyPTIC Consortium (2018). Prediction of susceptibility to first-line tuberculosis drugs by DNA sequencing. N Engl J Med.

[10] Olaru ID, Patel H, Kranzer K, Perera N (2018). Turnaround time of whole genome sequencing for mycobacterial identification and drug susceptibility testing in routine practice. Clin Microbiol Infect.

[11] Cabibbe AM, Trovato A, De Filippo MR, Ghodousi A, Rindi L, Garzelli C (2018). Countrywide implementation of whole genome sequencing: an opportunity to improve tuberculosis management, surveillance and contact tracing in low incidence countries. Eur Respir J.

[12] Zürcher K, Reichmuth ML, Ballif M, Loiseau C, Borrell S, Reinhard M (2021). Articles mortality from drug-resistant tuberculosis in high-burden countries comparing routine drug susceptibility testing with whole-genome sequencing: a multicentre cohort study. Lancet Microbe..

[13] Heyckendorf J, Andres S, Köser CU, Olaru ID, Schön T, Sturegård E (2018). What is resistance? Impact of phenotypic versus molecular drug resistance testing on therapy for multi- and extensively drug-resistant tuberculosis. Antimicrob Agents Chemother.

[14] Grobbel HP, Merker M, Köhler N, Andres S, Hoffmann H, Heyckendorf J (2021). Design of multidrug-resistant tuberculosis treatment regimens based on DNA sequencing. Clin Infect Dis.

[15] Park M, Satta G, Kon OM (2019). An update on multidrug-resistant tuberculosis. Clin Med J R Coll Phys Lond..

[16] WHO treatment guidelines for isoniazid-resistant tuberculosis Supplement to the WHO treatment guidelines for drug-resistant tuberculosis. 30285343

[17] Satta G, Edmonstone J (2018). Consolidation of pathology services in England: have savings been achieved?. BMC Health Serv Res.

[18] Nimmo C, Shaw LP, Doyle R, Williams R, Brien K, Burgess C (2019). Whole genome sequencing *Mycobacterium tuberculosis* directly from sputum identifies more genetic diversity than sequencing from culture. BMC Genomics.

[19] Brown AC, Bryant JM, Einer-Jensen K, Holdstock J, Houniet DT, Chan JZM (2015). Rapid whole-genome sequencing of *Mycobacterium tuberculosis* isolates directly from clinical samples. J Clin Microbiol.

[20] Doyle RM, Burgess C, Williams R, Gorton R, Booth H, Brown J (2018). Direct whole-genome sequencing of sputum accurately identifies drug-resistant mycobacterium tuberculosis faster than MGIT culture sequencing. J Clin Microbiol.

[21] Swai HF, Mugusi FM, Mbwambo JK (2011). Sputum smear negative pulmonary tuberculosis: sensitivity and specificity of diagnostic algorithm. BMC Res Notes.

[22] Rapid Diagnostic Technologies: Status and Limitations - The Global Crisis of Drug-Resistant Tuberculosis and Leadership of China and the BRICS - NCBI Bookshelf. https://www.ncbi.nlm.nih.gov/books/NBK195973/

[23] The use of molecular line probe assays for the detection of resistance to isoniazid and rifampicin.

[24] Rapid Communication: Molecular assays as initial tests for the diagnosis of tuberculosis and rifampicin resistance. 2020. http://apps.who.int/bookorders.

[25] Eddabra R, Ait BH (2018). Rapid molecular assays for detection of tuberculosis. Pneumonia..

[26] Deeplex Myc-TB. https://www.genoscreen.fr/en/genoscreen-services/products/deeplex

[27] WHO. Technical guide on next-generation sequencing technologies for the detection of mutations associated with drug resistance in Mycobacterium tuberculosis complex. https://www.who.int/publications/i/item/WHO-CDS-TB-2018.19

[28] Cabibbe AM, Spitaleri A, Battaglia S, Colman RE, Suresh A, Uplekar S (2020). Application of targeted next-generation sequencing assay on a portable sequencing platform for culture-free detection of drug-resistant tuberculosis from clinical samples. J Clin Microbiol.

[29] Mokrousov I, Chernyaeva E, Vyazovaya A, Sinkov V, Zhuravlev V, Narvskaya ON (2016). Next-generation sequencing of *Mycobacterium tuberculosis*. Emerg Infect Dis.

[30] WHO consolidated guidelines on tuberculosis. Module 3: diagnosis—rapid diagnostics for tuberculosis detection 2021. 34314130

